# Supplementary figures and images for: The GalNAc-T Activation (GALA) Pathway: Drivers and markers
Source: PLoS One. 2019 Mar 19;14(3):e0214118. doi: 10.1371/journal.pone.0214118 (PMC6424425; doi:10.1371/journal.pone.0214118)

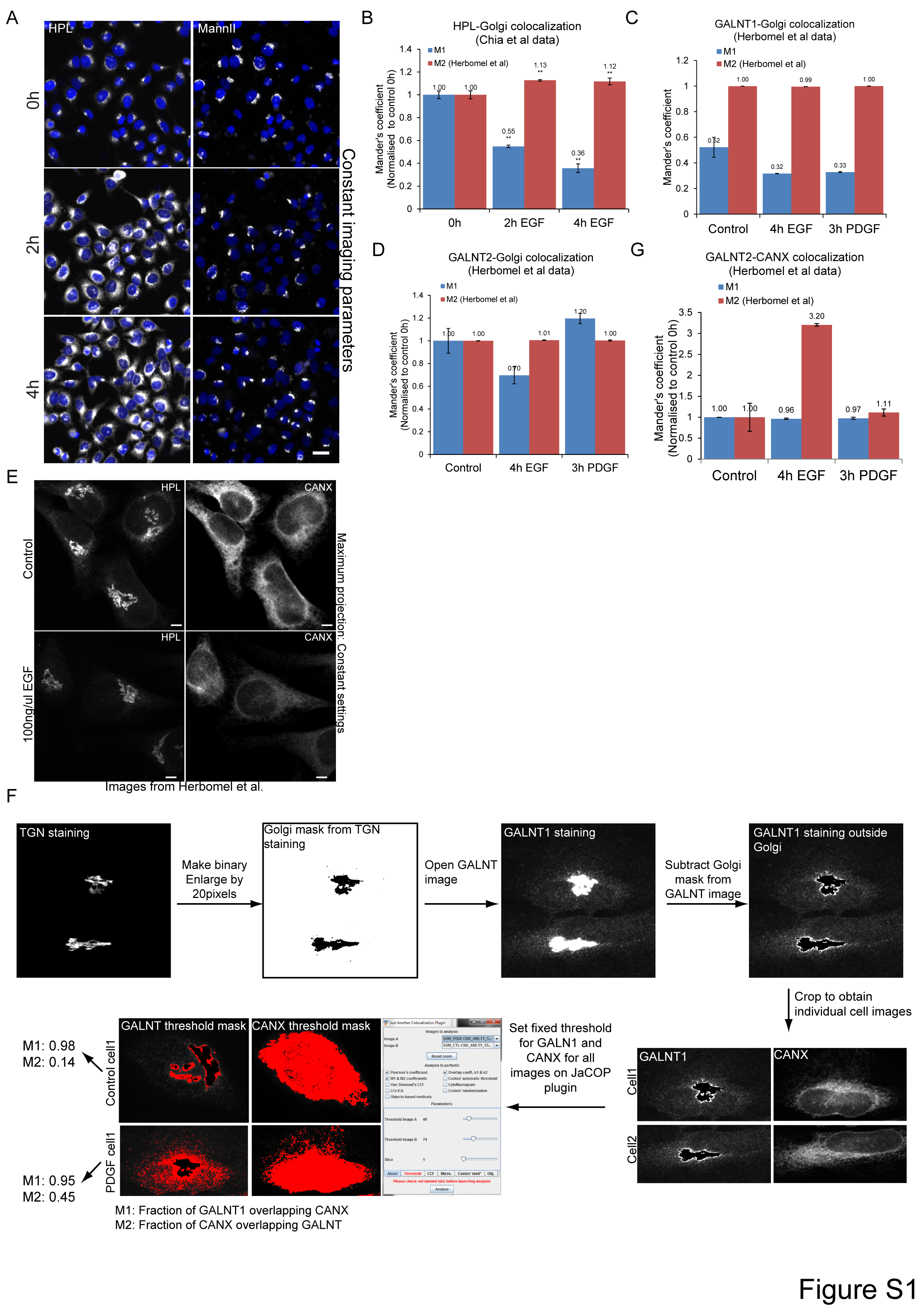

Supplement: S1 Fig — (A) HPL staining of cells stimulated with EGF over the indicated durations performed in Bard’s lab. The Golgi is demarcated by MannII-GFP. Scale bar: 30 μm. (B) Mander’s coefficient to quantify the level of colocalization between HPL and Golgi marker MannII-GFP over time of EGF stimulation in (A). M1 represents the fraction of HPL staining overlapping the Golgi and M2 represents the fraction of Golgi overlapping HPL staining. Values were normalised with respect to unstimulated control cells (0 h). Hundreds of cells were quantified. Statistical significance (p) measured by two-tailed paired t test. **, p < 0.001 relative to unstimulated cells (0 h). (C) Mander’s coefficient values of GALNT1 and Golgi marker TGN in Fig 1F. (D) Quantification of Mander’s coefficient of GALNT2 and Golgi marker TGN in representative images provided by Tabak’s group. (E) Maximum projection from representative images stained with HPL and ER marker CANX provided by Herbomel et al. Scale bar: 5 μm. (F) Workflow on ImageJ to remove Golgi localised GALNT signal to quantify the extent of relocated GALNT with ER marker. See materials and methods section for more details. (G) Quantification of Mander’s coefficient of GALNT1 and ER marker CANX after removal of Golgi localized GALNT2 staining. M1 represents the fraction of GALNT1 staining coincident with the ER and M2 represents the fraction of the ER marker coincident GALNT2 staining. (TIF) [file pone.0214118.s001.tif]

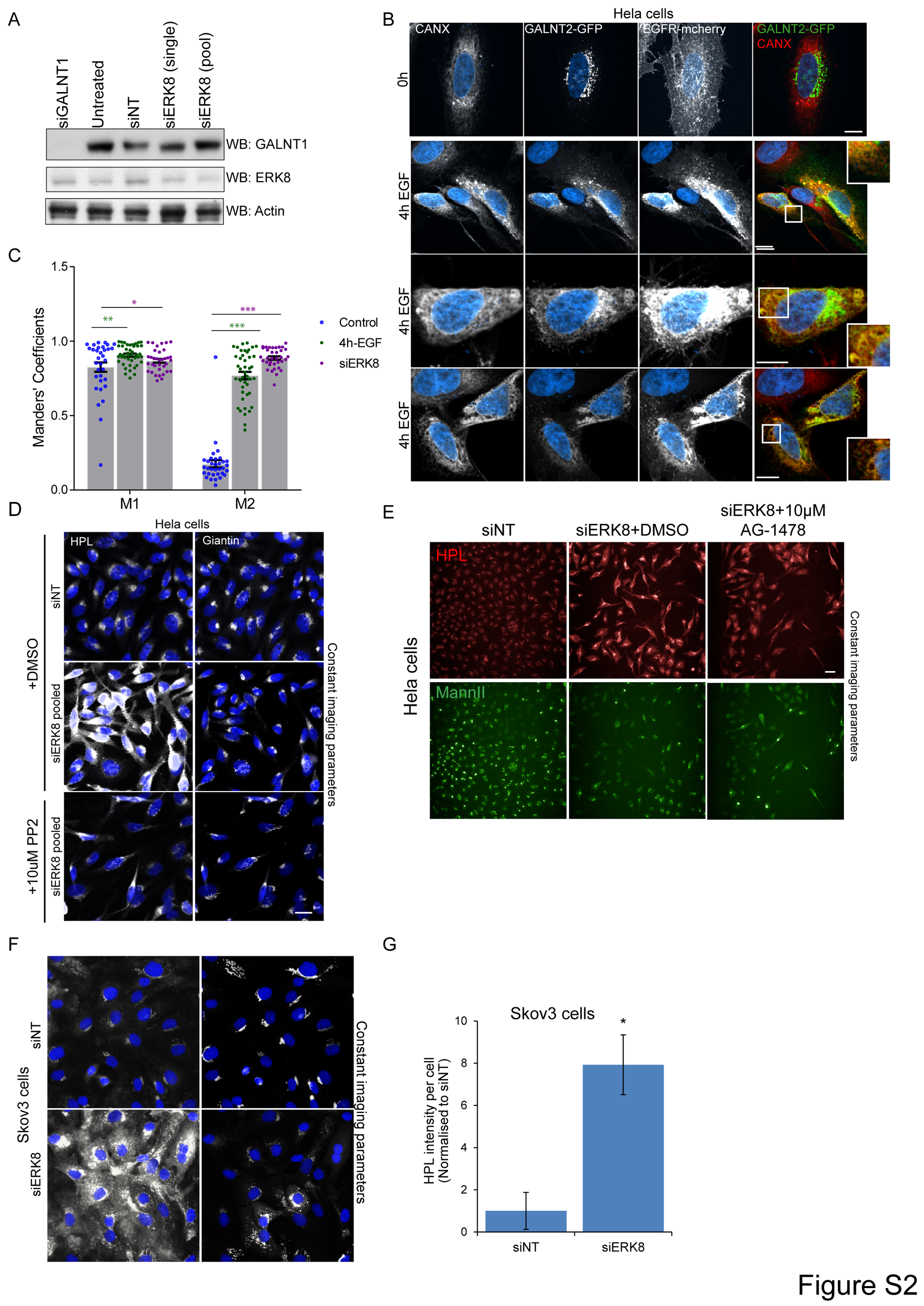

Supplement: S2 Fig — (A) Immunoblot analysis of GALNT1 levels in Hela cells depleted with ERK8 single (“siERK8 (single)) or ERK8 pooled (“siERK8 (pooled)) siRNA. (B) More representative images of GALNT2-GFP cells expressing EGFR-mcherry with and without EGF stimulation. Scale bar: 10 μm (C) Quantification of Mander’s coefficient quantification in EGFR expressing GALNT2-GFP cells. More than 33 cells were quantified for each condition. Statistical significance (p) measured by two-tailed paired t test. *, p < 0.05, **, p < 0.01 ***and p < 0.001 relative to unstimulated cells (0 h). (D) HPL staining of ERK8 depleted Hela cells treated with DMSO control, 10 μM Src inhibitor PP2 or 10 μM Src Kinase Inhibitor I (SKI-I) for 24 hours. Scale bar: 30 μm (E) HPL staining of ERK8 depleted Hela cells (“siERK8”) treated with 10 μM EGFR inhibitor AG-1478 or DMSO control. Scale bar: 30 μm. (F) HPL staining of ERK8 depleted Skov-3 cells. Scale bar: 30 μm. (G) Quantification of HPL intensity in (F). Statistical significance (p) measured by two-tailed paired t test.*, p < 0.05 relative to siNT control. (TIF) [file pone.0214118.s002.tif]
